# The modified south African triage scale system for mortality prediction in resource-constrained emergency surgical centers: a retrospective cohort study

**DOI:** 10.1186/s12913-017-2541-4

**Published:** 2017-08-23

**Authors:** Jacques Massaut, Pola Valles, Arnold Ghismonde, Claudinette Jn Jacques, Liseberth Pierre Louis, Abdulmutalib Zakir, Rafael Van den Bergh, Lunick Santiague, Rose Berly Massenat, Nathalie Edema

**Affiliations:** 1 Nap Kenbe Hospital Haiti, Médecins Sans Frontières OCB, Université Libre de Bruxelles, Rue Antoine Bréart 90, 1060 Brussels, Belgium; 2 Medical Department, Médecins Sans Frontières OCB, Rue de l’Arbre Bénit 46, 1050 Brussels, Belgium; 3 Nap Kenbe Hospital Haiti, Médecins Sans Frontières OCB, Tabarre, Rue La Fleur, Tabarre, Port-au-Prince, Haiti; 4 Médecins Sans Frontières OCB, Quala-e-Fatullah, Street #3, House #4, Kunduz, Kabul District 10 Afghanistan; 5 Operational Research Unit, Médecins Sans Frontières OCB, Rue de l’Arbre Bénit 46, 1050 Brussels, Belgium

**Keywords:** Emergency department, Limited resource setting, Prognostic model, Triage

## Abstract

**Background:**

The South African Triage Scale (SATS) was developed to facilitate patient triage in emergency departments (EDs) and is used by Médecins Sans Frontières (MSF) in low-resource environments. The aim was to determine if SATS data, reason for admission, and patient age can be used to develop and validate a model predicting the in-hospital risk of death in emergency surgical centers and to compare the model’s discriminative power with that of the four SATS categories alone.

**Methods:**

We used data from a cohort hospitalized at the Nap Kenbe Surgical Hospital in Haiti from January 2013 to June 2015. We based our analysis on a multivariate logistic regression of the probability of death. Age cutoff, reason for admission categorized into nine groups according to MSF classifications, and SATS triage category (red, orange, yellow, and green) were used as candidate parameters for the analysis of factors associated with mortality. Stepwise backward elimination was performed for the selection of risk factors with retention of predictors with *P* < 0.05, and bootstrapping was used for internal validation. The likelihood ratio test was used to compare the combined and restricted models. These models were also applied to data from a cohort of patients from the Kunduz Trauma Center, Afghanistan, to validate mortality prediction in an external trauma patients population.

**Results:**

A total of 7618 consecutive hospitalized patients from the Nap Kenbe Hospital were analyzed. Variables independently associated with in-hospital mortality were age > 45 and < = 65 years (odds ratio, 2.04), age > 65 years (odds ratio, 5.15) and the red (odds ratio, 65.08), orange (odds ratio, 3.5), and non-trauma (odds ratio, 3.15) categories. The combined model had an area under the receiver operating characteristic curve (AUROC) of 0.8723 and an AUROC corrected for optimism of 0.8601. The AUROC of the model run on the external data-set was 0.8340. The likelihood ratio test was highly significant in favor of the combined model for both the original and external data-sets.

**Conclusions:**

SATS category, patient age, and reason for admission can be used to predict in-hospital mortality. This predictive model had good discriminative ability to identify ED patients at a high risk of death and performed better than the SATS alone.

**Electronic supplementary material:**

The online version of this article (doi:10.1186/s12913-017-2541-4) contains supplementary material, which is available to authorized users.

## Background

Emergency triage is the systematic process of determining the priority for treatment based on the severity of the patients’ conditions. The principal aim of triage is to ensure that patients receive the most appropriate level of care according to their clinical status, while focusing attention on patients at a higher risk of death [1]. Identifying patients at a high risk of death is important in the emergency department (ED) to offer adequate treatment and to recognize patients in need of more intensive management and possible admission to an intensive care unit (ICU). Triage is recognized as a central component of the ED and was first introduced in the 1950s in the USA [2, 3]. More recently, the need for triage systems was also identified in low-resource settings with reports showing that the process of triage can improve patient flow, reduce patient waiting times, and decrease mortality in these contexts [4, 5]. These contexts can be characterized by few health resources including a limited number of physicians or qualified nurses and/or limited drugs or health materials.^1^ As one of the only validated tools that exists for the triage of patients in low-resource settings, Médecins Sans Frontières (MSF) has chosen the South African Triage Scale (SATS) [6–8] as a standard tool for its EDs such as Nap Kenbe Surgical Center in Tabarre, Port-au-Prince (Haiti) and in Kunduz (Afghanistan) [9, 10]. The SATS uses a physiology-based scoring system, the Triage Early Warning Score (TEWS), and a list of discriminators designed to triage patients into one of four color-coded priority groups for medical attention. Physiological variables used to compute the TEWS are mobility, temperature, systolic blood pressure, heart rate, respiratory rate, and neurological status. The TEWS also depends of the presence of trauma. Patients with a TEWS >6 are distributed into the red group, a TEWS of 5 to 6 into the orange group, a TEWS of 3 to 4 into the yellow group, and a TEWS of 0 to 2 into the green group. A fifth group (blue) allows to deal with the patients who looks obviously dead at admission. Discriminant parameters can move a patient into a higher priority group. Convulsions, burns on the face, and hypoglycemia < 55 g/dL were criteria to include patients in the red group; high-energy transfer, non-controlled hemorrhage, acute dyspnea, hemoptysis, thoracic pain, open fracture, member dislocation, member ischemia, post-epilepsy coma, focal neurological deficit, alteration of consciousness, burns over 20% of the body, burns from electricity or chemicals, circumferential burns, intoxication, and overdose were criteria for inclusion in the orange group; and controlled hemorrhage, closed fracture, burns over less than 20% of the body, finger or toe dislocation, and abdominal pain were criteria for inclusion in the yellow group. More specific discriminants are used under the age of 13 years. Following triage, patients in the red group must be managed immediately before patients from the other groups according to their priority. Gottschalk reported a detailed description of the SATS first know as the Cape triage score that contains more details and a SATS score table [8].

The aim of our study was to verify if data from the SATS system combined with other easily available patient characteristics can facilitate the identification of patients at high risk of mortality. Such patients could then receive more focused supportive care during their inpatient stay. For this purpose, we constructed and validated a prognostic model based on information available from the ED, including the reason for admission according to the MSF classification (Table 1) and data from the SATS system, and compared the model’s discriminative power with that of a model based on the four categories of the SATS alone. We hypothesized that the combined model would allow for better identification of patients at higher risk of in-hospital mortality, as it would take into account other independent factors linked to mortality. That model could be useful for audit purpose but also for clinical purpose as it would focuses attention on underestimated factors linked to mortality.

**Table 1:** Characteristics of patients in the emergency department who were admitted to Nap Kenbe Hospital, Haiti, or to Kunduz Hospital, Afganistan

	Nap Kenbe Hospital, Haiti	Kunduz Hospital, Afganistan
Age (median)	27	20
Gender (Male)	75%	83%
Reason for admission		
Burns	6 (0.1%)	1 (0.05%)
Traffic and road accidents	3070 (40%)	601 (28.22%)
Assault	74 (1%)	39 (1.83%)
Gunshot wound	991 (13%)	443 (20.8%)
Knife wound	510 (7%)	39 (1.83%)
Blast	0	294 (13.80%)
Mine	0	23 (1.08%)
Torture	41 (1%)	0
Other trauma (work, sport, and domestic accidents, etc.)	2356 (31%)	690 (32.39%)
Non-trauma (peritonitis, obstruction, etc.)	551 (7%)	0
Other (rare causes)	19 (0.2%)	0
Total admissions	7618	2130
SATS category		
Red	617 (8%)	563 (26%)
Orange	3106 (41%)	1087 (51%)
Yellow	3601 (47%)	466 (21%)
Green	294 (4%)	4 (0.2%)
Mortality	2.2%	4.9%

## Methods

### Study design

This was a retrospective cohort study for the development and validation of a model of in-hospital death prediction.

### Setting and study site

The study was performed in the emergency medicine, anesthesia, and intensive care departments of the Nap Kenbe Hospital: a 121-bed tertiary surgical center run by MSF in Tabarre, Port-au-Prince, Haiti. This MSF center, a pre-fabricated modular hospital, started providing specialized care for trauma and acute surgical conditions in February 2012 to cover the post-earthquake gap in trauma care.

Data from the surgical center run by MSF in Kunduz, Afghanistan were used as an external data-set. MSF had been working in Kunduz since August 2011 when the Kunduz Trauma Center was opened. This trauma center was fully functioning as a hospital at the time it was destroyed by bombardment on October 3, 2015.

### Study population

**Fig. 1:**
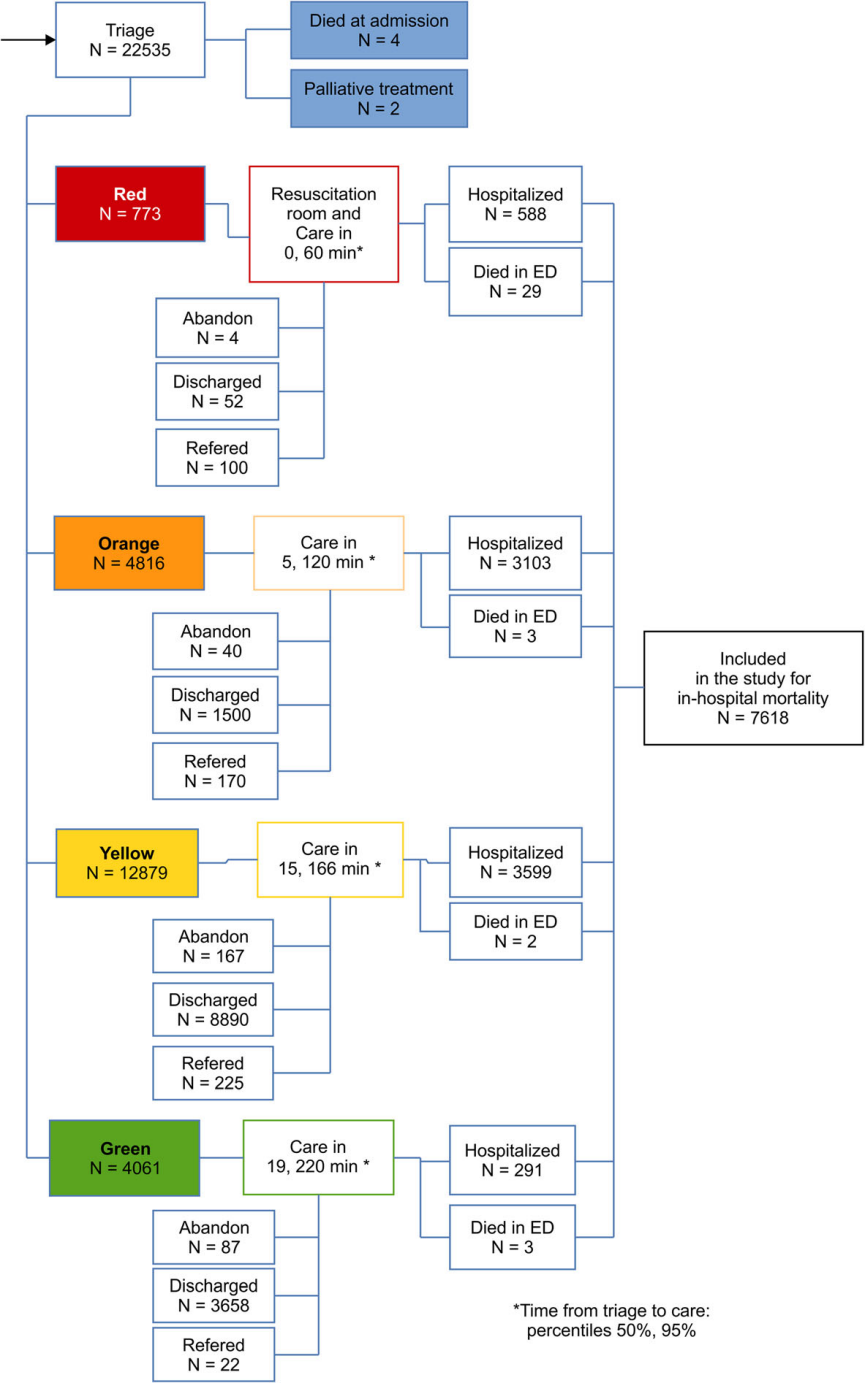
A flowchart from triage to hospitalization and time to care in the emergency department of the Nap Kenbe Hospital is shown

This study included all consecutive patients hospitalized at the Nap Kenbe Hospital from the ED between January 2013 and June 2015 (Additional file 1: Table S1). Admission criteria at the Nap Kenbe Hospital included patients presenting acute trauma or an acute surgical condition requiring hospitalization with the exceptions of isolated head, ocular, or spine trauma, obstetrical/gynecological cases, and burn cases. Patients that did not meet the admission criteria were stabilized in the ED on arrival if needed, and then referred to other institutions, if possible. For the external data-set from Kunduz Hospital, the study included all consecutive patients hospitalized between November 2014 and August 2015(Additional file 2: Table S2). The admission criteria at Kunduz Hospital were less restrictive for trauma cases, as patients with isolated head trauma were also admitted, but the admission criteria did not include non-trauma patients. At both hospitals, patients who died in the ED were considered as hospitalized. Patients discharged home from the ED or referred to other institutions from the ED were not considered for inclusion as they were not hospitalized, or were hospitalized at other institutions and did not contribute to our in-hospital mortality. However these patients where discharged from the ED after the triage score was applied and some of theses patients do go on to die outside of hospital after discharge or in another hospital. However we had no follow-up data for these patients and they could not be included. Patients declared dead on arrival (four cases at Nap Kenbe Hospital) and patients considered for palliative care at admission (two cases at Nap Kenbe Hospital) were also excluded from the study. The flow chart in Fig. 1 describes the triage process on arrival for patients in the ED at Nap Kenbe Hospital. Patient triage was performed at the patient entrance to the ED to evaluate priorities in care and treatment, but not to decide whether to discharge the patient to their home or transfer the patient to other institutions. Abandon was defined as patient refusal to be treated inside the hospital. The time from triage to care was shortest in the red category (0 min for the 50th percentile and 60 min for the 95th percentile) followed by the orange category (5 and 120 min), yellow category (15 and 166 min), and green category (19 and 220 min).

### Data sources

All data from patients arriving in the ED of the Nap Kenbe Surgical Center were routinely collected in the department’s paper register and encoded in Excel spreadsheets (Microsoft, Redmond, WA, USA). Data collected in the Excel files included age, sex, time of admission, reason for admission divided into nine groups according to the MSF classification, SATS triage category, and interventions like tracheal intubation, cardiopulmonary resuscitation, or thoracic drainage performed in the ED. Age, sex, reason for admission, and triage category were retrospectively extracted from the Excel files and used as candidate parameters in the analysis of factors associated with mortality. In seven cases, data were missing from the electronic records and were therefore retrieved from the patients’ charts. The TEWS was not routinely registered in the electronic database and thus could not be reasonably considered as a candidate parameter for analysis in this retrospective study of more than 7000 patients.

For the data-set from Kunduz Hospital, only electronic databases stored outside the hospital could be used, as all electronic records at Kunduz and all patients’ charts were destroyed in the October 3, 2015 bombardment of the hospital. For these reasons, we could only recover data for consecutive admitted patients from November 2014 to August 2015.

### Statistical methods

We used chi-squared and Mann-Whitney tests as appropriate to determine uni-variate associations with mortality. *P*-values <0.05 were considered significant. A multivariate logistic regression was used to isolate independent risk factors, compute odds ratios and regression coefficients, and predict the probability of death during the hospital stay. For the selection of factors, a stepwise backward elimination was performed, with the retention of predictors with *P* < 0.05. The Brier score was used to estimate the model’s performance. The model’s discriminative ability was measured as the area under the receiver operating characteristic (AUROC) curve. Bootstrapping was used for internal validation. The validation procedure was performed by sampling with replacements within each of 300 permuted data-sets of the same size as the original. Model optimism values computed from bootstrapping were used to correct the Brier score and the AUROC of the final model. We also applied the models to data from a cohort of patients from the Kunduz Trauma Center, Afghanistan, to validate mortality prediction in an external trauma patients population. The comparison of the discriminative powers of the combined model and the model restricted to the four SATS categories was performed using the likelihood ratio test. For the statistical analysis, we used Stata 8.2 (StataCorp, College Station, TX, USA).

## Results

At the Nap Kenbe Hospital from January 1, 2013 to June 30, 2015, there were 7618 patients hospitalized from the ED. The male sex predominated (75%) and the mean age of the patients was 29 ± 17 years, with ages ranging from 0 to 102 years. The most frequent reasons for admission were trauma-related lesions due to traffic and road accidents (3070, 40%), work, sport, and domestic accidents (2356, 31%), gunshot wounds (991, 13%), wounds from knives (510, 7%), and non-traumatic causes (e.g. acute surgical conditions such as peritonitis or obstructions) (551, 7%). Of the 7618 admitted patients, 171 (2.22%) died during hospitalization (Table 1). Thirty-seven of these patients died in the ED, 25 in the operating room, and 104 (56% of all deaths) in the ICU. The classification of all hospitalized patients according to the SATS, including those who died in the ED or during hospitalization, was 617 (8%) in the red category, 3106 (40%) in the orange category, 3601 (47%) in the yellow category, and 294 (4%) in the green category. Multivariate analysis identified the red and orange categories of the SATS, non-traumatic reasons for admission, age between 45 and 65 years and age over 65 years as independent factors associated with mortality, and these were retained in the final combined model (Table 2). No trauma category was retained in the multivariate regression analysis as an independent factor linked to mortality. The score of the combined model can be computed using the coefficients resulting from the logistic regression:

$$ \mathrm{Score}=4.17{6}^{\ast}\mathrm{R}+1.26{6}^{\ast}\mathrm{O}+1.14{8}^{\ast}\mathrm{NT}+0.71{1}^{\ast}\mathrm{Age}45\_65+1.64{0}^{\ast}\mathrm{Age}65-5.918, $$

where R is 1 if the patient is in the red category and 0 otherwise, O is 1 if the patient is in the orange category and 0 otherwise, NT is 1 for non-trauma patients and 0 otherwise, Age45_65 is 1 if the patient is older than 45 and lower or equal to 65 years and 0 otherwise, Age65 is 1 if the patient is older than 65 and 0 otherwise. The probability of death can be computed using the following formula:

$$ \mathrm{Predicted}\kern0.34em \mathrm{Death}\kern0.34em \mathrm{Rate}={\mathrm{e}}^{\left(\mathrm{Score}\right)}/\left(1+{\mathrm{e}}^{\left(\mathrm{Score}\right)}\right). $$

**Table 2:** Final combined model odds ratios and coefficients predicting in-hospital mortality among Nap Kenbe Hospital patients

Factor	Odds Ratio	95% CI-OR	Coefficients	95% CI-Co	*P* value
Red	65.079	37.703, 112.334	4.176	3.629, 4.721	< 0.001
Orange	3.546	1.988, 6.325	1.266	0.687, 1.845	< 0.001
Non-trauma	3.152	1.869, 5.315	1.148	0.626, 1.671	< 0.001
Age > 45 and < =65 years	2.036	1.343, 3.085	0.711	0.295, 1.126	< 0.001
Age > 65 years	5.154	2.843, 9.346	1.640	1.045, 2.235	< 0.001
Intercept			−5.918	−6.442, −5.394	< 0.001

The combined model divided the population into 18 groups with increasing probabilities of death according to the combination of parameters in the final combined model (Table 3).

**Table 3:** Predicted and observed mortality among patients admitted to the Nap Kenbe Hospital, Haiti

Group	Predicted mortality	Observed mortality	Red	Orange	Non-trauma	Age > 45 < = 65 years	Age > 65 years	N
1	0.0027	0.0033	0	0	0	0	0	3012
2	0.0054	0.0073	0	0	0	1	0	412
3	0.0084	0	0	0	1	0	0	269
4	0.0095	0.0066	0	1	0	0	0	2409
5	0.0137	0.0073	0	0	0	0	1	137
6	0.017	0.0196	0	0	1	1	0	51
7	0.0191	0.0256	0	1	0	1	0	390
8	0.0292	0.0336	0	1	1	0	0	149
9	0.0419	0.0625	0	0	1	0	1	16
10	0.0469	0.0608	0	1	0	0	1	115
11	0.0577	0.0937	0	1	1	1	0	32
12	0.1342	0.2222	0	1	1	0	1	9
13	0.149	0.167	1	0	0	0	0	497
14	0.2628	0.1875	1	0	0	1	0	80
15	0.3557	0.2381	1	0	1	0	0	21
16	0.4744	0.3333	1	0	0	0	1	15
17	0.5291	1	1	0	1	1	0	3
18	0.7399	1	1	0	1	0	1	1

### Internal validation and performance of the combined model

**Fig. 2:**
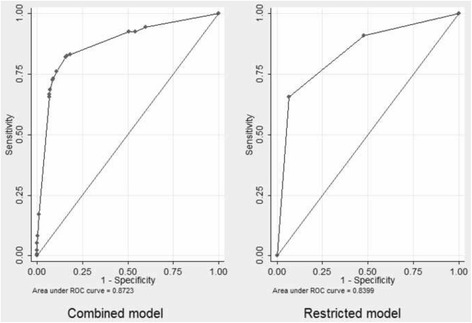
Receiver operating characteristic curves for the final combined mortality prediction model and the restricted model from the Nap Kenbe trauma center. The areas under the curves are 0.87 for the combined and 0.83 for the restricted model

The AUROC of the final model was 0.8723, showing good discriminative ability (Fig. 2). The bootstrap optimism estimate was 0.0122 (95% confidence interval [CI]: 0.011, 0.013). The AUROC of the final model corrected for optimism was 0.8601, showing good internal validity. All parameters retained in the final model had *P-*values <0.05 in over 90% of the bootstrap samples.

The Brier score corrected for optimism was 0.0195, suggesting good calibration of the model. The bootstrap optimism estimate was 0.0009 (95% CI: 0.0009, 0.0010). The model fitted the data well (Table 3) with no significant difference between the observed and the predicted mortality (*P* = 0.12, Pearson chi square).

### External data-set

From November 2014 to August 2015, there were 2130 patients hospitalized at the surgical center run by MSF in Kunduz. That cohort of patients had an in-hospital mortality rate that was significantly higher than that in Tabarre (4.9% versus 2.2%, *P <* 0.001) and was characterized by a higher proportion of red cases (47% versus 8%, *P* < 0.001). The mean age of the Kunduz patient population was also lower (23 ± 16 years versus 29 ± 17 years, *P* < 0.001). The combined model run on that external data-set had an AUROC of 0.834 (Fig. 2), a Brier score of 0.041 and accurately predicted the mortality rate (4.96% predicted versus 4.93% observed).

### The SATS risk estimate

**Fig. 3:**
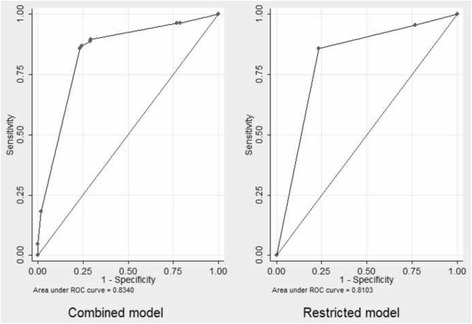
Receiver operating characteristic curves for the final combined mortality prediction model and the restricted model from the Kunduz trauma center. The areas under the curves are 0.83 for the combined and 0.81 for the restricted model

For the Nap Kenbe Hospital population, the risk estimate model based only on the four SATS categories showed inferior performance compared with the performance of the combined model. Only the red and orange categories were retained as independent factors linked to mortality, as mortality in the yellow and green categories were not statistically different. The overall calibration and discriminative ability were good (Brier score, 0.0197; AUROC, 0.8398) (Fig. 3). The optimism calculated from the bootstrap validation for the AUROC was 0.0126 (95% CI: 0.1158, 0.0136). The optimism for the Brier score was 0.0010 (95% CI: 0.0009, 0.0010). The likelihood ratio test comparing the combined model with the model using SATS estimation alone was highly significant in favor of the combined model (*P* < 0.0001).

For the Kunduz Hospital population, the restricted model overestimated mortality (5.6% predicted versus 4.93% observed mortality). The AUROC was 0.8103 (Fig. 3) and the Brier score 0.0426. For the external population, the likelihood ratio test comparing the combined model with the model using SATS estimation alone was also highly significant in favor of the combined model (*P* = 0.0001).

## Discussion

In this retrospective cohort study, we illustrated how routine ED data, such as information from the SATS system combined with the reason for admission and age of the patient can be used to predict in-hospital mortality. The described model showed good discrimination in identifying patients at a higher risk of in-hospital death. The SATS is a triage system that is easy to use and implement, and was demonstrated to have been successfully implemented and used in resource-constrained settings outside South Africa [9, 10]. Our combined model allows for the identification of patients meriting enhanced monitoring and supportive care during their inpatient stay because of an increased risk of death.

The use of routinely available parameters to assess mortality risk is one of the main strengths of our study, leading to low rates of missing data. The sample size of the cohort of analyzed patients was large enough to offer good statistical power. The combined model was internally validated using the bootstrapping technique, which is the most widely recommended for internal validation as it allows derivation of the final model from the full derivation sample and does not waste precious information by developing the model from a random part of the original data-set, as with the splitting method [11, 12]. The combined model was also applied on an external data-set, accurately predicted in-hospital mortality in the external population, and had better prognostic performance than the model restricted to the four SATS categories for both populations. However, the entire model could not be validated on that external population of only trauma patients.

Different risk scoring systems for the triage of emergency patients have been described [13–19], but their performances outside the contexts and settings used in their validations cannot be warranted [20]. Furthermore, several of these models were designed to be used in a non-surgical ED and cannot be used in our surgical settings. The discriminative power of the combined model presented in this study, with an AUROC corrected for optimism of 0.8601, compares well with the powers of models described in the previous literature. The AUROCs of these models ranged from 0.65 to 0.90 for the best performing model, which has an optimism-corrected AUROC of 0.90 [19], or with the inclusion of blood test results [13]. Our model is more adapted to resource-constrained settings and does not rely only on complex computations to predict the risk of death for every patient, as the data from Table 3 can simply be used for this purpose.

The surgical characteristics of the patients in the cohort limit the usefulness of the model to surgical facilities. Another limitation concerns the proportion of trauma versus non-trauma cases in the studied population, where trauma cases were more frequent. Caution is needed when applying the model to populations of other centers, as the results from the study may depend on the nature of the emergency surgical center and the proportion of trauma versus non-trauma cases in the treated population. The exclusion of patients discharged from the ED or transferred to others institutions in our study may also have influenced the results in some extend.

## Conclusions

In conclusion, in this cohort study of 7618 surgical patients, we found that a prediction model derived from SATS system data, combined with the reason for admission and age of the patient, all readily available after the triage of patients at arrival in the ED, was a good predictor of the subsequent risk of death. Such a model is well adapted to use in resource-constrained surgical settings, predicted well mortality when applied to an external data-set of trauma patients, and performed better than a model based only on the SATS categories.

## Additional files

Additional file 1: Table S1. Contents the entire data-set of the cohort of patients hospitalized at the Nap Kenbe Hospital Haiti that served to establish the model. (CSV 252 kb)

Additional file 2: Table S2. Contents a simplified data-set of both Nap Kenbe Haiti and Kunduz Afganisthan that served to apply the model on the cohort of Kunduz patients. The patients from Kunduz have the value 1 for the kunduz variable. (CSV 150 kb)

## Abbreviations

AUROC: Area under the receiver operating characteristic curve
CI: Confidence interval
ED: Emergency department
ICU: Intensive care unit
MSF: Médecins Sans Frontières
SATS: South African Triage Scale
TEWS: Triage early warning score

## Footnotes

1 In the case of the Nap Kenbe Hospital, health resources were limited by the absence of intra-hospital facilities including a computed tomography scanner, angiography, autologous blood recovery, dialysis, bacteriology, and neurosurgery; difficulties in obtaining fresh frozen plasma and platelet concentrates; and limitations in laboratory tests including only electrolytes, hemograms, urea and creatinine levels, some coagulation tests, and blood gas analyses. The possibilities in the nine-bed ICU were also limited by the availability of only four ventilators and the absence of invasive monitoring. In cases requiring computed tomography or dialysis, the patient must be transported to other institutions for these procedures.
